# Distribution, genetic polymorphism and genotype prediction of Rhesus blood group antigens among the Kurdish population of Zakho, Kurdistan Region, Iraq

**DOI:** 10.1371/journal.pone.0338158

**Published:** 2026-04-16

**Authors:** Shakir A. Zebari, Sawer S. Ahmed, Ibrahim A. Naqid, Shivan U. Muhammed, Abdullah S. Muhi

**Affiliations:** 1 Department of Biomedical Sciences, College of Medicine, University of Zakho, Zakho, Kurdistan Region, Iraq; 2 Medical Laboratory Technology, College of Health and Medical Technology, Duhok Polytechnic University, Duhok, Kurdistan Region, Iraq; 3 Department of Clinical Sciences, College of Medicine, University of Zakho, Zakho, Kurdistan Region, Iraq; German Red Cross Blood Service, GERMANY

## Abstract

The Rhesus blood group system exhibits significant polymorphism, with diverse antigen distributions across populations. This study investigates the antigenic profile, haplotype frequencies, and genotype predictions in a cohort from Zakho, Kurdistan Region, Iraq, which is crucial for transfusion medicine and genetic studies. A prospective cross-sectional analysis was performed on 1,000 Kurdish individuals at Zakho Emergency Teaching Hospital. Blood samples were phenotyped for Rh (D, C, c, E, e) antigens. Haplotype assignments were made using Fisher-Race terminology, and probable genotypes were calculated based on allele frequencies and Wiener nomenclature. The most common Rhesus blood antigens are Rh(e) (95%), Rh(D) (91.9%), Rh(C) (76%), and Rh(c) (68%). Rh(E) is found in 25.8% of individuals, while only 8.1% are Rh(D) negative. Among the Rh(D) positive population, the most frequent phenotype/haplotype and presumed genotype was DCe (*DCe/DCe, R1R1*) at 31.4%, followed by DCce (*DCe/dce, R1r*) at 29.3%, and DCcEe (*DCe/DcE, R1R1*) at 13.8%. The dce phenotype (*dce/dce, rr*, in 7.2%) was the most common among Rh(D) negative individuals. No significant differences were observed between sexes. This study reveals that DCe, DCce, and DCcEe are prevalent phenotypes among Rh(D) positive individuals, whereas the dce haplotype predominates among Rh(D) negative individuals. The Rhesus phenotype/genotype aligns with Kurdish and Arab groups in Iraq and shows partial resemblance to Western European, Indian, and Iranian populations, but significantly differs from African-American populations, except for the dce phenotype (*dce/dce, rr)*. These findings are crucial for blood transfusion strategies, donor selection, maternal and fetal health, the prevention of Rh incompatibilities, and genetic research in the region.

## Introduction

The Rhesus (Rh) blood group system is the second most frequently considered blood group system in clinical practice, following the ABO system [1]. The Rh blood group is encoded by two tightly linked loci located on chromosome 1; specifically, the *RHD* gene encodes the RhD antigen, while the *RHCE* gene encodes the RhCE antigens. A sequence of unknown significance known as *SMP1* separates the *RHD* and *RHCE* genes [2]. The Rh blood group system exhibits considerable complexity, as various antigens arise from combinations of single-nucleotide variations and gene rearrangements [3].

This blood group system comprises 55 antigens, which are carried on two proteins, RhD and RhCE, each consisting of 417 amino acids. However, only five antigens (D, C, c, E, and e) are recognised as clinically significant. Among these, the Rh (D) antigen is noteworthy due to its highly immunogenic properties, leading to extensive research and testing. This is largely attributed to its association with hemolytic transfusion reactions [4]. The lack of compatibility testing for Rh blood groups may result in the development of antibodies during transfusion; the recipient’s immune system produces such antibodies in response to foreign Rh antigens present on transfused red blood cells (RBCs). These immune responses can precipitate serious clinical complications, including acute hemolysis, organ dysfunction, or fetal morbidity in Rh-negative individuals [5].

Numerous studies in Iraq have documented the distribution of ABO and Rh(D) blood groups among Kurdish and Arab populations [6,7]. Data on the frequencies of other Rh antigens (C, c, E, e) are scarce. The present study was conducted to evaluate the prevalence, phenotypic distribution, and genotypic prediction of the Rh blood group system within the Kurdish population residing in Zakho City, Kurdistan Region of Iraq.

## Materials and methods

This prospective cross-sectional study was conducted at a premarital screening centre at Zakho Emergency Teaching Hospital between January 1^st^ 2021, and January 31^st^ 2023. The study included the first five couples who attended mandatory premarital screening daily, with a total of 1000 participants included in this study.

Non-Kurdish populations and non-residents of Zakho City were excluded from this study.

Ethical Approval was obtained from the Research Ethics Committee, College of Medicine/ University of Zakho. Issued on the 10^th^ of December 2020. Reference number: DEC2020/UOZE35.

Verbal informed consent was obtained from all participants. Sociodemographic data were collected, including names, dates of birth, residency, and phone numbers. These records were stored and kept in confidential files and accessed only by authorised personnel.

Three millilitres (ml) of blood were collected from each participant by proper phlebotomy techniques. The blood was distributed into a K3-Ethylene-Diamine-Tetraacetic-Acid (EDTA) vacutainer tube and well mixed on a rotatory mixer.

The Rh(D) antigen status of all participants was determined using the standard slide agglutination technique with BIOSCOT CE-marked IgM monoclonal Anti-D IgM (clone RUM-1) reagent (Bioscot Ltd., United Kingdom, distributed in the U.S. and Canada), which directly agglutinates normal D and most weak/partial D types but does not agglutinate D category VI red cells. Additionally, phenotyping for the other Rh antigens (C, c, E, and e) was performed using the conventional tube agglutination method with the corresponding BIOSCOT® CE-marked IgM monoclonal antisera (anti-C, anti-c, anti-E, and anti-e), from Bioscot Ltd. All procedures were conducted in strict accordance with the manufacturer’s instructions. A positive and negative control provided by the manufactures are used with each batch of the reagents to ensure test validity.

Phenotype frequencies of the five main Rh antigens (D, C, E, c, and e) were calculated by dividing the count of positive each antigen by the total number of individuals screened, with results expressed as a percentage. Phenotypes and genotypes were designated according to Fisher's race/ Wiener’s nomenclature. Precise genotype determination is unfeasible without testing family members or conducting DNA analysis; thus, the most probable genotype is inferred from gene frequency estimates.

Descriptive statistical methods were employed utilising IBM SPSS Statistics, version 27, to elucidate the findings of the study, including a Chi-square test of independence, with a p-value threshold of less than 0.05 deemed statistically significant.

## Results

A total of 1,000 participants were enrolled, comprising 500 males (50%) and 500 females (50%). Age was approximately normally distributed. The mean age was 25.69 ± 6.04 years, with a median of 25.4 years (range: 18–55 years). The consanguinity rate among couples (third and fourth-degree relatives) was 28.5%.

The overall prevalence of Rh(D) positive was found in 919 (91.9%) of subjects, while 81 (8.1%) were found to be Rh(D) negative. No statistically significant difference in Rh(D) distribution was observed among males and females, “Table 1”.

**Table 1:** Distribution of Rh(D) antigen status by sex among the studied population.

Sex	Rh(D) Positive n (%)	Rh(D) Negative n (%)	Total n (%)	*p-value
**Female**	460 (92.0)	40 (8.0)	500 (50)	0.91
**Male**	459 (91.8)	41 (8.2)	500 (50)	
**Total**	919 (91.9)	81 (8.1)	1000 (100)	

*p-value is calculated using the chi-square test of independence

Variable distribution of other Rh blood antigens was observed among the studied population. Rh(e) antigen was present in 960 (96%), followed by Rh (C) antigen in 761 (76.1%) and Rh(c) antigen in 680 (68%), while Rh (E) antigens were the least prevalent and were only positive in 258 (25.8%). No statistically significant difference in Rh (e, C, c, E) antigen distribution was observed among males and females, “Table 2”.

**Table 2:** Distribution of other Rh blood group antigens by sex among the studied population.

Rh Phenotype	Overall Positive n (%)	Male n (%)	Female n (%)	*p-value
**Rh(e)**	960 (96.0%)	481 (96.2%)	479 (95.8%)	0.75
**Rh(C)**	761 (76.1%)	381 (76.2%)	380 (76.0%)	0.94
**Rh(c)**	680 (68.0%)	340 (68.0%)	340 (68.0%)	1
**Rh(E)**	258 (25.8%)	128 (25.6%)	130 (26.0%)	0.88

*p-value is calculated using the chi-square test of independence

In this study, the most prevalent Rh phenotypes/ Haplotypes were DCe (31.4%), DCce (29.3%), and DCcEe (13.8%). These were followed by DcEe (8.0%), dce (7.2%), Dce (5.4%), and DcE (3.3%). Less frequent phenotypes/Haplotypes included DCcE (0.7%), dCe (0.6%), and Cce (0.3%). A variety of phenotypes, notably (DCEe, DCE, CcEe, CcE, CE, cEe and cE) were not detected “Table 3”.

**Table 3:** Frequency of Rh blood group antigens and corresponding Fisher-Race haplotypes/phenotype distribution among the studied population.

^*^Reaction pattern with antisera					Fisher–Race haplotype / Phenotype	Number of Cases (n)	Percentage (%)
D	C	c	E	e			
+	+	–	–	+	DCe	314	31.4
+	+	+	–	+	DCce	293	29.3
+	+	+	+	+	DCcEe	138	13.8
+	–	+	+	+	DcEe	80	8
+	–	+	–	+	Dce	54	5.4
+	–	+	+	–	DcE	33	3.3
+	+	+	+	–	DCcE	7	0.7
–	–	+	–	+	ce	72	7.2
–	+	–	–	+	Ce	6	0.6
–	+	+	–	+	Cce	3	0.3
Total						1000	100

* (+) indicate positive reaction, (-) indicate negative reaction.

When these results are stratified by Rh(D) status, it is observed that among Rh(D) positive samples, the most common phenotypes/haplotype are DCe (34.2%), DCce (31.9%), DCcEe (15%), followed by DcEe (8.7%), Dce (5.9%), DcE (3.6%), and DCcE (0.8%). Reflecting the predominant haplotype distribution in Rh(D+) individuals. While among Rh(D) negative samples, the dce haplotype was the most predominant (88.9%), followed by dCe (7.4%) and Cce (3.7%), highlighting the dominance of the dce haplotype among Rh(D–) individuals.

In this study, the most probable genotypes among Rh(D) positive individuals were *DCe/DCe (R₁R₁,* 34.2%), *DCe/dce (R₁r,* 31.9%), and *DCe/DcE (R₁R₂,* 15.0%). Less frequent genotypes included *DcE/dce (R2r*, 8.7%), *Dce/dce (R0r*, 5.9%), *DcE/DcE (R₂R₂,* 3.6%), and *DCE/DcE (RzR₂,* 0.8%) “Table 4”. Among Rh(D) negative individuals, the predominant genotype was *dce/dce (rr,* 88.9%), followed by *dCe/dce (r’r,* 7.4%) and *dCe/dCe (r’r’*, 3.7%) “Table 4”.

**Table 4:** Fisher-Race haplotype/Phenotype distribution, Genotype Prediction, most probable genotype and Wiener Nomenclature by Rh(D) Status among the studied population.

Rh(D) Status	Fisher–Race Haplotype / Phenotype	%	Possible Genotypes (haplotype pairs)	^#^Most Probable Genotype	^$^Wiener Nomenclature
^*^ **Rh(D) positive n=919**	DCe	34.2	*DCe / DCe or DCe / dCe*	*DCe / DCe*	*R1R1*
	DCce	31.9	*DCe / dce or DCe / Dce*	*DCe / dce*	*R1r*
	DCcEe	15	*DCe / DcE or DCe / dcE or Dce / DCE or Dce / Dce*	*DCe / DcE*	*R1R2*
	^*†*^DcEe	8.7	*DcE / dce or DcE / Dce or dcE/Dce*	^*†*^ *DcE / dce*	^*†*^ *R2r*
	Dce	5.9	*Dce / dce or Dce / Dce*	*Dce / dce*	*R0r*
	DcE	3.6	*DcE / DcE or DcE / dcE*	*DcE / DcE*	*R2R2*
	DCcE	0.8	*DCE / DcE or DCE / dcE*	*DCE / DcE*	*RzR2*
^**^ **Rh(D) negative n=81**	ce (dce)	88.9	*dce / dce*	*dce / dce*	*rr*
	Cce	7.4	*dCe / dce*	*dCe / dce*	*r'r*
	Ce (dCe)	3.7	*dCe / dCe*	*dCe / dCe*	*r'r'*

*Rh(D)-positive haplotypes: contain at least one D allele.

**Rh(D)-negative haplotypes: lack the D allele.

^†^For DcEe, both *DcE/dce (R2r)* and *DcE/Dce (R2R0)* are possible; *R2r* is considered more frequent.

^#^The “Most Probable Genotype” is determined based on allele frequency in general populations.

^$^Wiener nomenclature: standardised to ensure compatibility with serological and molecular studies.

## Discussion

The study of blood group systems provides insights into human evolution and diversity. Analysing variations among populations helps trace migrations, reveal genetic relationships, and understand adaptations over time. Additionally, knowledge of blood group distribution is crucial in clinical medicine, genetic research, and anthropology, linking biology to our shared heritage.

Blood transfusions are essential for saving lives but carry risks. The immune system may produce alloantibodies when exposed to incompatible red blood cells, leading to reactions that can range from mild to severe, including hemolytic disease in fetuses and hemolytic reactions post-transfusion [8].

Antibodies against clinically significant antigens, like Rh antigens, can cause hemolytic transfusion reactions and hemolytic disease in newborns. These complications may be encountered routinely in clinical practice. Recognising these complications is essential in clinical practice, and comprehensive phenotypic detection of these antigens can help in their prevention and management [9].

The Rh antigen is inherited in a codominant manner via the autosomal chromosome 1, suggesting no significant sex differences. However, due to the Kurdish population's diverse religious groups (Muslim, Christian, and Yezidi), comparative analyses were performed to account for variations in Rh antigen polymorphisms linked to religious differences.

The current study revealed that the distribution of the Rh(D)positive antigen was 91.9%. This finding aligns with that from a previous study in Iraq-Duhok [6,10], suggesting genetic homogeneity among Kurdish populations in northern Iraq, and with that of the African-American [11]. However, the observed prevalence is lower than that recorded in China [12], and it is slightly higher than the prevalence found within the Iraqi-Arab, Iranian, Indian and Western European descent population [7,11,13–15] “Table 5”.This may be due to geographical variation and ethnic background of the population. Using monoclonal Anti-D IgM (clone RUM-1) reagent from Bioscot, detect and directly agglutinates normal D, weak D and most D partial types; however, it fails to agglutinate D category VI red cells unless an IgM/IgG blended reagent in indirect agglutination or automated method is used. So, examining the presence and prevalence of this variant form of the D antigen could be a focus of future research in this region.

**Table 5:** Comparative frequencies (%) of Rh antigens among different populations.

Population / Region	Rh(D)+	Rh(D)−	Rh(C)	Rh(c)	Rh(E)	Rh(e)
**Iraq (Duhok**) [6]	91.1	8.9	75.9	68.1	25.1	95.6
**Iraq (Basrah**) [7]	88.8	11.2	68.3	77.7	52.6	96.7
**Western European descent** [11]	85	15	70	80	30	98
**African-American** [11]	92	8	34	97	21	99
**China** [12]	99.4	0.6	88.77	53.63	44.94	92.61
**Iran** [13,15]	87.76	12.24	73.6	72.1	30.83	93.59
**India** [14]	84.35	15.65	81.74	56.32	21.74	100
**Current study**	91.1	8.9	76.1	68	25.8	96

The Rh(D) negative frequency in this study (8.9%) aligns closely with Iraqi-Duhok [6] and African-American [11], but is lower than reported from other global studies [7,11,13,14] except for that reported in China [12]. The similarity between Zakho and Duhok suggests genetic homogeneity among Kurdish populations in northern Iraq. In contrast, Basrah (southern Iraq, more Arab-majority) shows a slightly higher Rh(D) negative frequency, possibly reflecting historical gene flow from neighbouring Persian or South Asian populations. The low Rh(D) negative prevalence rate underscores the need for a D-negative blood inventory for emergency, obstetric, and transfusion services. However, without molecular genotyping, or using a blended IgM/IgG monoclonal antibody using indirect antiglobulin or an automated method, some individuals might be “Weak D IV” variants masked within the Rh-negative statistics.

The frequency of the Rh(e) antigen observed in this study is consistent with findings reported in other studies [6,7,11]. The e allele is recognised as the ancestral and most prevalent variant at the RHCE locus. Typically, frequencies of the Rh(e) antigen exceed 95% worldwide. Consequently, the significant prevalence of the Rh(e) antigen poses considerable challenges in identifying e-negative blood donors for patients with alloanti-e antibodies. Furthermore, the occurrence of anti-e as an autoantibody adds another layer of complexity to the provision of compatible blood, “Table 5”.

The prevalence of the Rh(E) antigen in this study is consistent with findings reported among Kurdish populations in Duhok, Iraq [6], and in certain regions of Iran [15], suggesting a limited frequency of E-carrying Rh haplotypes (e.g., DcE, dcE). However, it is higher than the frequencies reported in African-American and Indian people [11,14], and lower than those observed in Arab populations in Basra, Iraq [7], as well as in Chinese and Western European descent populations [11,12]. Despite its relatively low population prevalence, the Rh(E) antigen is highly immunogenic, second only to D among Rh antigens. Consequently, anti-E is among the most frequently encountered non-D Rh antibodies in multiply transfused patients. This may explain the higher rates of alloimmunization to E compared with the more prevalent but less immunogenic Rh(e) antigen “Table 5”.

In this study, the high frequency of the Rh(C) antigen mirrors that of Kurdish populations in Duhok, Iraq [6], Iran [15], and India [14], suggesting shared Indo-Iranian or ancient Mesopotamian genetic influences. Furthermore, there is some degree of resemblance to the Iraqi-Arab and Western European descent population [7,11]. However, the levels observed in our study are lower than those documented for the Chinese [12] and significantly higher than those found in the African-American population [11] “Table 5”.

Regarding the Rh(c) antigen, our study shows similarity to that reported from Kurdish Iraqis [6] and to some extent with the Iranians [15] and slightly lower than the Iraqi Arabs (Basrah) [7], possibly indicating regional substructure within Iraq. While it is quite higher than reported by the Chinese and Indian [12,14], and lower than reported from Western European descent and African-American [11], “Table 5”.

This study evaluated Rh phenotype frequencies based on observed antigen expression patterns and established genotype–phenotype correlations from previous population studies. Direct genotyping was not conducted; probable genotype assignments were made following documented allelic associations from earlier population studies [11].

The most frequent Rh haplotype observed among the Rh(D)-positive Kurdish population in this study was DCe (R₁), followed by Dce (R₀) and DcE (R₂), corresponding to the presumed genotypes *R₁R₁, R₁r*, and *R₁R₂,* with frequencies of 31.4%, 29.3%, and 13.8%, respectively. Among the Rh(D)-negative individuals, the most prevalent haplotype was *ce* (*r*), corresponding to the *rr* genotype, which accounted for 7.2% of the total studied population “Table 6”.

**Table 6:** Comparative frequencies of common Rh genotypes in different populations.

Rh(D) Status	Wiener/ Fisher-Race	Iraq (Duhok) [6]	Western European descent [11]	African-American [11]	Iranian [15]	Indian [14]	Current Study
**Positive**	*R1R1 (DCe/DCe)*	31.5	18.5	3	28.23	40.87	31.4
	*R1r (DCe/Dce)*	30.1	34.9	21	26.22	23.48	29.3
	*R1R2 (DCe/DcE)*	13.8	13.3	4	12.74	13.91	13.8
**Negative**	*rr (dce/dce)*	7.4	15.1	7	9.02	11.3	7.2

Data are presented as percentages.

The *R1R1* genotype exhibits a notably elevated frequency in the current study, aligning closely with previous findings reported from Duhok, Iraq [6]. This genotype's prevalence also shows some similarities with the data observed in the Iranian population [15]; however, it stands out significantly higher than the frequencies recorded among Western European and African-American groups [11]. In contrast, the occurrence of the *R1R1* genotype is slightly lower in Indian populations [14]. These patterns suggest the presence of a shared genetic heritage within the region, which may reflect a common ancestry among the populations studied or indicate limited genetic admixture with other groups where the *R1R1* genotype is less prevalent, such as those from African or European backgrounds. This phenomenon could potentially be explained by the high rate of consanguineous marriages prevalent within the Kurdish population in our region, as highlighted by this study (28.5%) and a previous study [16], which may contribute to the increased frequency of this particular genotype “Table 6”.

The *R1r* frequency in the current study is similar to that reported from the Duhok Iraqi population [6], indicating internal consistency. It is lower than in the Western European descendants [11] but higher than in African-American, Iranian and Indian groups [11,14,15]. Suggesting this genotype is stable, may be ancestral or selectively neutral across a wide geographic range, but less common in those with predominant African ancestry, “Table 6”.

The *R1R2* genotype is notably high in the Kurdish population, closely mirroring the broader Iraqi Duhok, the Western European descent and the Indian [6,11,14]; however, it is much higher than reported from African-American [11] and lower than the Iranian populations [15]“Table 6”.

The *dce/dce (rr,* 7.2%) genotype frequency reported in this study is consistent with global patterns of the Rh(D) negative population; however, it is among the lowest reported, similar to African-Americans and the Iraqi Kurdish population [6] and slightly lower than reported from Iran and India [14,15]. This contrasts sharply with the Western European descents, where Rh negativity is nearly twice as common [11]. The similarity with African-Americans may be coincidental, as the underlying haplotype structures differ. The lower Rh(D)-negative prevalence in Kurds implies reduced risk of hemolytic disease of the fetus and newborn (HDFN) compared to the Western European descent populations, but antenatal screening remains essential, “Table 6”.

## Conclusion

This study characterises the distribution of Rh antigens and phenotypes in the Kurdish population of Zakho, revealing a distinct regional pattern. The e, D, C, and c antigens were highly prevalent, whereas the E antigen was less frequent (25.8%), and 8.1% of individuals were Rh(D) negative. Among Rh(D)-positive individuals, DCe, DCce, and DCcEe were the most common phenotypes, while the ce haplotype predominated in Rh(D)-negative subjects.

The Rh phenotype/genotype aligns with Kurdish and Arab groups in Iraq and shows partial resemblance to the Western European descent, Indian, and Iranian populations, but significantly differs from African-American populations, except for the dce phenotype (*dce/dce, rr)*.

These findings highlight the clinical importance of extended Rh antigen profiling beyond routine ABO and Rh(D) typing. Limited screening for C, c, E, and e antigens may increase the risk of alloimmunization, particularly among multi-transfused patients. It is important to have a comprehensive understanding of local Rh antigen distribution as this knowledge is crucial for optimising blood bank inventory management, fostering the development of well-structured voluntary donor registries, and minimising the risk of alloimmunization. Additionally, implementing timely detection strategies and administering anti-D immunoglobulin can significantly reduce the challenges posed by hemolytic disease of the fetus and newborn.

Broader population-based studies across Kurdistan and Iraq are warranted to strengthen transfusion safety and maternal–fetal health outcomes.
